# Developing a novel welfare assessment tool for loose-housed laying hens – the Aviary Transect method

**DOI:** 10.1016/j.psj.2021.101533

**Published:** 2021-10-13

**Authors:** Guro Vasdal, Joanna Marchewka, Ruth C. Newberry, Inma Estevez, Kathe Kittelsen

**Affiliations:** ⁎ Norwegian Meat and Poultry Research Centre, Oslo 0513, Norway; † Institute of Genetics and Animal Biotechnology, Polish Academy of Sciences, Jastrzębiec, Magdalenka 05-552, Poland; ‡ Norwegian University of Life Sciences, Faculty of Biosciences, Department of Animal and Aquacultural Sciences, P.O. Box 5003, Ås 1432, Norway; § Neiker-Tecnalia, Arkaute Agrifood Campus, Animal Production, P.O. Box 46, Vitoria-Gasteiz E-01080, Spain; # IKERBASQUE, Basque Foundation for Science, 48009 Bilbao, Spain

**Keywords:** laying hen, aviary housing, animal welfare, welfare assessment methodology, transect sampling

## Abstract

This study compared welfare assessment results in aviary flocks using 3 approaches: 1) A novel Aviary Transect method, 2) AssureWel, and 3) the Norwegian farm advisors’ NorWel method. The Aviary Transect time requirement, interobserver reliability, and within- and across-house sensitivity to detect welfare indicators were also evaluated. The study was conducted on 6 randomly chosen commercial white-strain layer flocks of similar age and flock size, kept in multitiered aviaries. The Aviary Transect method comprised standardized walks along each aisle while screening the whole flock for 12 welfare indicators: feather loss (**FL**) on head, back, breast, and tail, wounds on head, back, tail, and feet, dirty birds, enlarged crop, sick birds, and dead birds. AssureWel involved scoring FL on head and back, and dirtiness of 50 random birds, and flock-level evaluation of beak trimming, antagonistic behavior, flightiness, birds needing further care, and mortality. NorWel involved scoring 8 welfare indicators on 50 random birds: FL on head, back, breast, and tail, dirtiness, and wounds on head, back, and tail. The AssureWel detected flock differences in both minor and major FL on the back (*P* < 0.01) as well as somewhat dirty birds (*P* < 0.01). The NorWel method detected flock differences in both minor and major FL on the head (*P* < 0.01), back (*P* < 0.001), breast (*P* < 0.001), and tail (*P* < 0.001) and somewhat (score 1) dirty birds (*P* < 0.05). The Aviary Transect method detected flock differences in FL on head, back, breast, and tail (all *P* < 0.001), dirty birds (*P* < 0.05) and enlarged crop (*P* < 0.001). More birds with FL on breast, and more dirty birds, were found in wall vs. central transects (*P* < 0.05). There was good interobserver agreement, except for dirty birds (*P* < 0.01), and positive correlations (*P* < 0.05) were identified between the Aviary Transect method and the other sampling methods for FL on head and back, and dirtiness. The three methods took similar time to complete (about 20 min/flock). In conclusion, all 3 methods detected significant differences in welfare indicator prevalence between flocks. The new Aviary Transect method provides egg producers with an efficient and sensitive whole-flock assessment of hen welfare status in multitiered aviaries.

## INTRODUCTION

An integral part of ethical and sustainable egg production is to ensure acceptable hen welfare. Non-cage housing systems, such as multitiered aviaries, are increasingly used in commercial egg production. These systems offer the birds more space and opportunities to perform natural behavior compared to enriched cages (Widowski et al., 2016). However, aviary systems can also pose welfare challenges for the hens, including a higher risk of poor plumage (Heerkens et al., 2015), damaging feather pecking (Lay et al., 2011), and mortality (Rodenburg et al., 2008), especially in non–beak-trimmed birds (Sepeur et al., 2015). Improving welfare is most important for the hens themselves, but is also an important competitive arena for producers and businesses. Increasingly, businesses are required to demonstrate that animal welfare requirements are being met, thus the pressing need for practical on-farm welfare assessment efforts. On-farm welfare assessment is a complex task because it should be noninvasive, comparable across flocks and houses, and cost-efficient (Marchewka et al., 2013). It must also be based on validated indicators and reliable methods.

Several methods have been developed to assess animal welfare in commercial flocks of laying hens, including Welfare Quality (Welfare Quality®, 2009), LayWel (Tauson et al., 2005; Blokhuis et al., 2007), and AssureWel (Main et al., 2012). The Welfare Quality poultry protocol consists of detailed scoring of several welfare indicators on a limited sample of birds, and the evaluation requires, on average, 7 h/farm (van Niekerk et al., 2012). The LayWel project aimed to evaluate laying hen welfare in different systems and developed a scoring system consisting of 3 main indicators: plumage condition of 6 body parts, pecking damage on the comb and rear body, and bumblefoot (Blokhuis et al., 2007). AssureWel was developed as a practical tool for commercial use, based on a simplification of LayWel, and is estimated to only take 15 min/flock (Main et al., 2012). The AssureWel scoring system includes 7 indicator types: feather loss (**FL**), dirtiness, beak trimming, antagonistic behavior, flightiness, birds needing further care, and mortality. The recommended sample size of scored birds is 50 birds for both LayWel and AssureWel, and there is good agreement in plumage scores between the methods (Decina et al., 2019). Decina et al. (2019) reported that LayWel plumage scoring took about 50 min/50 birds, while AssureWel plumage scoring took 30 min/50 birds. AssureWel was found to be easier to understand and implement by producers compared to LayWel (Decina et al., 2019). Finally, Norwegian egg producers and advisors have developed a list of welfare indicators to record when observing flocks of laying hens during advisory visits, called NorWel (unpublished). This method bears similarities to AssureWel, but also includes scoring of wounds on different parts of the body. The NorWel scoring system includes 8 indicators assessed on 50 random birds: FL on head, back, breast, and tail, dirtiness, and wounds on head, back, and tail, and takes about 20 min/flock.

Contrary to the Welfare Quality protocol, the LayWel, AssureWel and NorWel methods are based on observations from a distance, and do not require bird handling. In general, the capture and handling of hens may reduce biosecurity (EFSA, 2008), is stressful for the birds, and poses a risk for sampling bias, as healthier birds may escape capture (Kjaer et al., 2011; Marchewka et al., 2013). Bright et al. (2006) compared plumage scoring from 2-m away with plumage scoring on captured birds and found good agreement between the methods. The same was reported by Kjaer et al. (2011), who found good agreement between assessing plumage from 2 to 3 m away and on captured birds. These results show that reliable data can be obtained by observing hens from a distance, thereby avoiding capture stress. However, a potential weakness of some of the methods is the limited sample size (50 birds). Visual assessment and manual recording of individual data on every bird in the entire flock is considered too time consuming for practical application, but a larger sample than 50 birds would provide a more reliable estimate of the plumage condition of the flock (Bright et al., 2006).

If a particular welfare assessment method is to be applied in practice by the industry, the method needs to be time efficient, accurate, and repeatable. Previous studies have shown that the transect walk is a practical and reliable method for assessing animal-based welfare indicators on-farm in large flocks of broilers (Marchewka et al., 2013; BenSassi et al., 2019a,b), turkeys (Marchewka et al., 2015, 2019; Ferrante et al., 2019), and ducks (Abdelfattah et al., 2020). The transect method is based on line transect sampling methodology, a technique routinely used in ecological and wildlife studies to estimate animal biodiversity and abundance (Butler et al., 2007). In short, an assessor walks through the house along predetermined paths while counting the number of birds observed within predefined welfare indicator categories. The method requires no animal handling and allows for the visual assessment of the entire flock or a representative proportion of it (Marchewka et al., 2013, 2015). The transect method is similar to the daily flock checks conducted by producers and should, therefore, be easy to apply in laying hen flocks.

The transect method for broilers and turkeys evaluates between 11 and 13 welfare indicators, including lameness, wounds, FL, dirtiness, sick birds, and dead birds (Marchewka et al., 2013, 2015). These indicators were selected as they are considered critical to the welfare status of meat poultry, and have a major economic impact (Estevez, 2007). All indicators are scored on a binary scale, whereby the observer records all animals clearly fitting each defined welfare indicator. Compared to a method such as AssureWel or NorWel, in which every assessed bird is scored on a graded scale, the transect method focuses on the more severe cases of welfare issues. In this way, inter-observer reliability is improved (D'Eath et al., 2012; Main et al., 2012; Marchewka et al., 2013, 2015), surveillance time is optimized, and the risk of omitting birds is minimized. The transect method is used as a benchmarking tool for the turkey Welfare Certification WELFAIR in Spain which currently has certified over 75% of the national production, underlining the applicability of the method by advisors and auditors. An aviary transect method for evaluating important welfare problems in cage-free laying hens could, therefore, reap the benefits of whole flock assessment, providing an efficient, reliable, and quantitative assessment of the welfare status of the flock.

Before implementing an aviary transect method, several aspects of the method need to be investigated and verified, including time requirements, interobserver reliability, and sensitivity. The method should include evaluation of plumage condition, dirtiness, wounds, and mortality, as these are considered important indicators of laying hen welfare (Blokhuis et al., 2007; Rodenburg et al., 2008). These indicators have been reported to be scored similarly by different observers (Decina et al., 2019) and their frequencies vary in a manner that can be useful for identifying specific housing and management issues (Blokhius et al., 2007), which are important qualities for animal-based indicators of animal welfare (EFSA, 2012). Sensitivity is a measure of how well the method can detect differences in prevalence of welfare indicators across flocks (EFSA, 2012). The transect method is reported to detect small variations in indicator prevalence when compared to individual bird assessment and slaughter data in turkeys (Marchewka et al., 2015) and broilers (BenSassi et al., 2019a,b), and is thus, considered sensitive for these production types.

Another aspect is the distribution of birds within the house. A higher prevalence of broilers with welfare issues has been reported in wall transects compared to central transects (BenSassi et al., 2019a). Also, contrary to broilers and turkeys, hens in aviary systems can move both horizontally and vertically between the tiers. While resources such as perches, nest boxes, litter, feeders, and drinker lines are evenly distributed along the length of the house, they are unevenly distributed between vertical levels of the house. Therefore, depending on the time of day, variable proportions of the flock will be found on the different tiers and on the litter floor (Campbell et al., 2016a). Furthermore, hen distribution is influenced by vertical location preferences. For example, hens often prefer the highest perches (Newberry et al., 2001; Campbell et al., 2016b), and only use lower perches when the highest ones are filled (Odèn et al., 2002). For these reasons, when applying transects sampling to loose-housed laying hens, it is important that both horizontal and vertical locations of the house are evaluated.

We hypothesized that transect sampling is an efficient, sensitive method for practical use in the egg industry, providing producers with a quantitative assessment of the welfare status of their flocks. The aim of this study was to investigate the sensitivity of each of the 3 different approaches: a new Aviary Transect method, AssureWel and NorWel, to detect differences in welfare indicator prevalence between flocks of laying hens in aviaries. For the Aviary Transect method, we evaluated the time required, interobserver reliability, and within-house sensitivity. The NorWel method also allowed assessment of within-house sensitivity.

## MATERIALS AND METHODS

### Animals and Housing

The study was conducted between August and October 2020 on 6 commercial farms located in eastern Norway. The studied flocks (1 flock/farm) were randomly selected from the supplier lists of 2 different egg packing companies and were visited once between the ages of 70 to 75 wk (Table 1). Producers were contacted a few weeks before the visit, and participation in the study was optional. All flocks consisted of approximately 7,500 white-strain hens (Dekalb White, n = 4; Lohmann LSL, n = 2) with intact beaks, housed in indoor multitiered aviary systems. The flocks were managed according to standardized practices with regards to feed, water, ventilation, litter, and lighting (KSL, 2020). The pullets arrived at the farm at around 16 wk of age and were kept until 78 wk when they were depopulated following standard commercial practices for Norway.

**Table 1:** Hybrid, flock size, animal density, aviary layout and time spent on the Aviary Transect assessment of 6 flocks.

Flock^1^	Hybrid	Age at visit (wk)	Flock size (n)	Floor area (m^2^)	Usable area (m^2^)^2^	Animal density (birds/m^2^)^3^	Aviary type	Light intensity (mean lux)	Transects (n)	Total flock assessment time (min)
1	Dekalb	71	7,500	1,000	1,850	4.05	Big Dutchman	11	4	19
2	Lohmann	70	7,700	506	990	7.77	Landmeco	6	4	25
3	Dekalb	72	7,840	432	915	8.56	Big Dutchman	5	3	19
4	Lohmann	74	7,200	385	815	8.83	Victorsson	7	2	20
5	Dekalb	71	7 500	648	1005	7.46	Landmeco	8	3	16
6	Dekalb	75	7,500	450	910	8.24	Landmeco	5	4	18

1 One flock/house and farm.

2 Usable area: an area at least 30 cm wide with a floor slope not exceeding 14%, with headroom of at least 45 cm. Nesting areas are not regarded as usable areas (EU Council Directive 1999/74/EC).

3 Animal density: number of birds/m^2^ of usable area.

**Figure 1:**
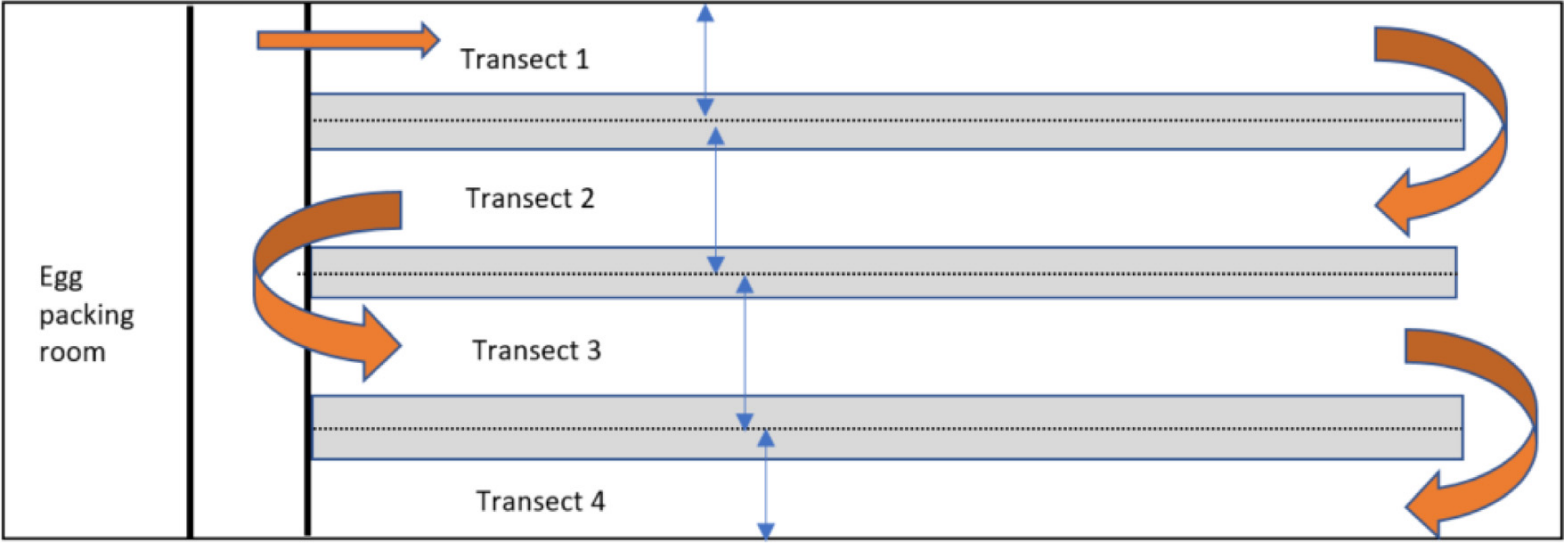
Schematic overview of a hen house (2-dimensional horizontal view, not to scale) showing 3 aviary structures (gray), transect width (blue arrows, dotted lines) and an example of a path taken by observers (orange arrows).

All flocks were housed in fully enclosed houses in one of 3 types of aviary systems, with automatic mechanical ventilation and artificial lighting. Mean light intensity ranged from 5 to 11 lux between houses (Table 1) as measured with a luxometer (Extech LED meter LT40, FLIR Commercial Systems Inc., Nashua, NH). The three aviary systems had similar layout, with 3 tiers above the floor, feed, and water lines on tiers 1 and 2, nest boxes on tier 2, and perches on tier 3. The houses were about 12 m wide, with wood shavings litter covering a floor area ranging from 385 m^2^ to 1,000 m^2^ that extended around and under the tiered aviary structures. Each aisle within the aviary designated a different transect. There were 1 to 3 rows of tiered structures along the length of the house, with a wall transect along each side, and up to 2 central transects, for a total of 2 to 4 transects in the different houses (Table 2; Figure 1).

**Table 2:** The width of each transect observed during transect walks in each flock, and estimated number of birds observed in each transect (T1 to T4).

Flock	Transects (n)	Aisle 1 width (m)^1^	Aisle 2 width (m)^2^	Aisle 3 width (m)^3^	Aisle 4 width (m)^4^	House width (m)	Structure width (m)	Flock size (n)	Birds/T1 (n)^1^	Birds/T2 (n)^2^	Birds/T3 (n)^3^	Birds/T4 (n)^4^
1	4	1.75	17.5	1.75	1.75	14.0	2.33	7,500	1,563	2,188	2,188	1,563
2	4	1.54	1.51	15.1	1.54	12.0	1.96	7,700	1,619	2,231	2,231	1,619
3	3	1.11	1.11	-	1.11	11.0	3.83	7,840	2,158	3,524	-	2,158
4	2	2.11	-	-	2.11	10.0	5.78	7,200	3,600	-	-	3,600
5	3	1.03	1.10	-	1.04	12.0	4.41	7,500	2,023	3,447	-	2,030
6	4	1.40	1.49	14.9	1.40	11.0	1.74	7,500	1,548	2,202	2,202	1,548

1 By left wall of house (relative to entrance door).

2 Central left of house.

3 Central right of house.

4 By right wall of house.

### Data Collection

Because the study did not involve any animal handling, experimental manipulations or invasive procedures, it was exempt from approval of animal use by the Norwegian Food Safety Authority (Norwegian regulations on use of animals in research, 2015). The study protocol stated that if a hen in a flock was observed to be suffering, the producer would be called immediately, and the hen would be humanely culled. Two observers with extensive poultry experience (G.V., K.K.) conducted the assessments. Before data collection started, the 2 observers visited 4 laying hen flocks together to practice the Aviary Transect methodology and achieve a high level of agreement in scoring. Data collection was then conducted on the 6 farms recruited to the study. Visits started around 0900 h. Each visit began with an explanation to the producer of the project goals and data collection procedure. Flock and house information, including house dimensions, was obtained at this time. After entering the house, the observers measured the width of the aisles and tiered structures. They then collected data using the Aviary Transect, AssureWel, and NorWel welfare assessment methods, in that order.

### Aviary Transect Method

Following the method of Marchewka et al. (2015), standardized transect walks were made along the full length of the house to record the number of hens observed per transect that were showing each of 12 predefined welfare indicators (Table 3). These indicators were selected as they are known to be critical for the welfare status of laying hens (Blokhuis et al., 2007; Rodenburg et al., 2008). The aisle width of each transect was measured with a laser measurer (Bosch Zamo II) from the wall to the aviary structure (for wall transects) or between 2 aviary structures (for central transects; Figure 1). The transect area assessed during each transect walk comprised the littered floor area in the aisle as well as half the width of the space under the aviary structure, and on each tier of the structure, on one side of each wall transect, and on both sides of each central transect. One observer started with the left wall transect (Transect 1) and the other with the right wall transect (Transect 4; Figure 1). Both observers started from the end of the house closest to the entrance door. When reaching the other end of the house, the observers returned collecting data in a different transect. This process was repeated in houses with more than 2 transects, so that all transects in the house were walked by both observers at different times, with the order followed by each observer balanced across houses. In houses with 3 transects, one observer would start with Transect 1, then Transect 4, and finally Transect 2. The other observer would start with Transect 4, then 2, and finally 1. The times for the start and end of the Aviary Transect assessment were noted down. The transect method took between 16 and 25 min to complete observations of the whole house, depending on the flock (Table 1).

**Table 3:** Description of 12 welfare indicator categories assessed by the Aviary Transect method.

Indicator^1^	Description
FL head	Missing feathers on the head, including the neck, ≥5 cm in diameter
FL back	Missing feathers on ≥50% of the back, including the wings
FL breast	Missing feathers on the breast, ≥5 cm in diameter
FL tail	Missing or clearly damaged feathers on the tail, mainly shafts and rachises left
Dirty	Prominent dark staining of the back, wing, or tail feathers, covering at least 25% of the body; not including light discoloration of feathers from dust.
Wounds head	Prominent marks on the head and neck, due to fresh or older wounds.
Wounds back	Prominent marks on the back, including the wings, due to fresh or older wounds.
Wounds tail	Prominent marks on the tail due to fresh or older wounds.
Wounds feet	Includes bumblefoot (visible dorsally), and prominent marks on the feet due to fresh or older wounds
Enlarged crop	Pendulous crop hanging in front of the breast
Sick	Clear signs of impaired health, including a small and pale comb, red-watery eyes, disarranged feathers, missing or deformed body parts, and clearly different (pale or yellowish) skin color; often found in a resting position
Dead	Dead bird found when walking along a transect

Abbreviation: FL, feather loss.

1 Hens could be classified as belonging to more than one category.

The observers moved slowly through the flock to minimize disruption of the birds during scoring. While walking along each transect, stops were made as needed to allow assessment of birds on the floor underneath the aviary, and on all three tiers. Birds in nest boxes were observed by opening approximately half of the nest box curtains. To observe birds on the top tier, the observers used steps or platforms on the side of the structure. To estimate the number of birds in each transect, the total number of birds in the house was divided by the width of transect, assuming that birds were homogeneously distributed throughout the length and width of the house (Table 2).

### AssureWel

Following the transect observations, one observer assessed 50 random birds in 6 different locations according to the AssureWel protocol (for parameter definitions, Table 4). Birds from a range of locations in the house such as the litter area, slatted area, perches and different tiers were assessed for FL on the head and back, and dirtiness (Table 4). While the observer moved around the house to score the 50 birds, the whole flock was observed and, in accordance with the AssureWel method, the following descriptive information was collected: beak trimming (yes or no), antagonistic behavior (number of incidents observed), flightiness of the flock (scored as calm, cautious, or flighty), and number of birds needing further care (Table 4). Data on the mortality (%) of the previous and current flock were obtained from the producer. The AssureWel method took approximately 20 min/flock.

**Table 4:** Description of the 8 AssureWel indicators scored on 50 random birds per flock.

Indicator^1^	Description
FL head (50 birds)	Feather loss on head and neck, scored 0: no loss, 1: < 5 cm, 2: > 5 cm diameter
FL back (50 birds)	Feather loss on back and vent, scored 0: no loss, 1: < 5 cm, 2: > 5 cm diameter
Dirtiness (50 birds)	Dirt on plumage, scored 0: the bird is clean, 1: soiling of at least one area on the bird but no area > 5 cm diameter, 2: soiling > 5 cm diameter on one or more areas of the bird
Beak trimming (whole flock)	Scored as a: Flock not beak trimmed, or, based on farm records, beak trimmed before 10 days of age, or beak trimmed as an emergency procedure under veterinary advice, or b: Number of birds seen with more than 1/3 beak removed.
Antagonistic behaviour (whole flock)	Includes aggressive behaviour: fighting, and aggressive pecking at or chasing other birds, and injurious feather pecking: pulling out feathers, and pecking at wounds or vent. Bird behavior is observed and listened to for 1 min and during the rest of the time spent in the house. Number of incidents of antagonistic behaviour observed or heard is recorded, identifying, if possible, whether either aggressive behavior or injurious feather pecking are observed.
Flightiness (whole flock)	Flock scored as Calm: in general, the birds appear undisturbed by your presence or actively approach you; Cautious: in general, the birds are disturbed by your presence but do not appear actively alarmed; or Flighty: the birds appear actively alarmed by your presence
Birds needing further care (whole flock)	Number of any sick or injured birds found that would benefit from hospitalization or culling. Recorded, if possible, according to signs of sickness or injury: sick, loose droppings, skin lesions, eye problem, lameness, other.
Mortality (whole flock)	a) Mortality of previous flock; b) Mortality to date; c) Mortality to 40 wk (where applicable), based on farm records

Abbreviation: FL, feather loss.

1 Scored either on 50 random birds or at the whole flock level (Main et al., 2012).

### NorWel

After the AssureWel observations, the same observer assessed another 50 random birds according to the NorWel (unpublished) indicator descriptions (for details on scoring, see Table 5). Birds from a range of locations in each vertical level of each transect were assessed for FL on the head, back, breast, and tail, on a scale from 0 (no loss) to 2 (>5 cm diameter of bare skin visible or, for the tail, substantial feather damage; Table 5). The same 50 birds were also scored for dirtiness on a scale from 0 (clean) to 2 (substantial soiling over >50% of plumage), and presence of wounds on head, back, and tail on a binary scale (1: clearly visible, fresh, or older wound; Table 5). For each bird receiving a score of 1 or 2, the location in the house (transect and vertical level) was recorded. The NorWel method took around 20 min/flock.

**Table 5:** Description of the 8 NorWel indicators scored on 50 random birds per flock.

Indicator^1^	Description
FL head	Feather loss on head, scored 0: no loss, 1: < 5 cm, 2: > 5 cm diameter
FL back	Feather loss on back and wings, scored 0: no loss, 1: < 5 cm, 2: > 5 cm diameter
FL breast	Feather loss on breast, scored 0: no loss, 1: < 5 cm, 2: > 5 cm diameter
FL tail	Feather loss on tail, scored 0: no wear, 1: some wear, 2: substantial wear, only shafts and rachises left
Dirty	Plumage, scored 0: clean, 1: some dirt, 2: > 50 % of plumage dirty
Wounds head	Head, scored 0: no wounds, 1: clearly visible fresh or older wound
Wounds back	Back including wings, scored 0: no wounds, 1: clearly visible fresh or older wound
Wounds tail	Tail, scored 0: no wounds, 1: clearly visible fresh or older wound

1 Abbreviation: FL, feather loss.

### Statistical Analyses

For the Aviary Transect data, we used the data collected by each of the 2 observers in each transect to calculate the frequency of birds with a particular welfare indicator as a proportion of the total estimated number of birds in each transect type (wall vs. central). For the AssureWel and Norwell data, the proportion of 50 birds with scores 1 and 2 was calculated for each welfare indicator category, by the total number of birds scored in the flock (AssureWel) or by birds count per transect type and vertical level of the house (NorWel). We analyzed the data in SAS Version 9.3 (SAS Institute Inc. 2013) using 3 generalized linear models (PROC GLIMMIX) with binomial distribution, one model per welfare assessment method. Each welfare indicator evaluated within a welfare assessment method was analyzed using the method-specific model, where the response variable was a single welfare indicator. The Aviary Transect model included flock, observer and transect type (wall or central) as fixed factors. The AssureWel model included only flock as a fixed factor. The NorWel model included flock, transect type (wall or central), and vertical level (floor plus 3 aviary tiers) as fixed factors. The flock factor provided a measure of the sensitivity of each method across flocks, the observer factor was introduced to test the interobserver reliability of the Aviary Transect method and the transect type factor was introduced to evaluate the within-house sensitivity of the Aviary Transect method (horizontal) and NorWel method (vertical and horizontal). Least Square Means (**LSM**) differences were adjusted for multiple comparisons using the post-hoc Tukey test. Spearman correlations were calculated using the PROC CORR script in SAS 9.3 (SAS, 2013) to evaluate relationships between the 3 welfare assessment methods for all comparable welfare indicator categories. *P*-values < 0.05 were considered statistically significant.

## RESULTS

### Aviary Transect Method

The Aviary Transect method detected significant variation across the studied flocks with regards to prevalence of FL on the head, back, breast, and tail (all *P* < 0.001), dirty birds (*P* < 0.05) and enlarged crop (*P* < 0.001; Table 6). More birds with FL on the breast, and more dirty birds, were observed in wall transects compared to central transects (*P* < 0.05; Table 6). The results showed good interobserver agreement for all welfare indicators except dirty birds (*P* < 0.01; Table 6).

**Table 6:** Analysis of variance for the prevalence of welfare indicators (mean ± SE % of birds) across flocks, observers and transects according to the Aviary Transect method.

	Flocks (n)	FL head	FL back	FL breast	FL tail	Dirty	Wounds head	Wounds back	Wounds tail	Wounds feet	Enlarged crop	Sick	Dead
Flock													
1	1	0.81 ± 0.061^a^	1.15 ± 0.072^a^	0.58 ± 0.069^a^	1.63 ± 0.125^a^	0 ± 0^b^	0 ± 0	0 ± 0	0 ± 0	0 ± 0	0.08 ± 0.025^a^	0.04 ± 0.013	0.06 ± 0.032
2	1	0.09 ± 0.025^b^	0.44 ± 0.023^ab^	0.05 ± 0.025^b^	0.06 ± 0.026^b^	0.25 ± 0.046^a^	0 ± 0	0.01 ± 0.006	0.01 ± 0.006	0.01 ± 0.008	0 ± 0^b^	0.01 ± 0.007	0.03 ± 0.022
3	1	0.26 ± 0.063^b^	0.78 ± 0.203^ab^	0.07 ± 0.021^b^	0.34 ± 0.101^ab^	0.02 ± 0.013^b^	0 ± 0	0.01 ± 0.006	0 ± 0	0.01 ± 0.007	0.01 ± 0.007^b^	0.03 ± 0.017	0.01 ± 0.011
4	1	0.03 ± 0.018^b^	0.04 ± 0.012^c^	0 ± 0^b^	0.01 ± 0.009^b^	0.01 ± 0.008^b^	0.02 ± 0.011	0.01 ± 0.006	0 ± 0	0.01 ± 0.008	0 ± 0^b^	0 ± 0	0.02 ± 0.012
5	1	0.20 ± 0.043^b^	0.23 ± 0.063^b^	0.05 ± 0.019^b^	0 ± 0^b^	0.14 ± 0.016^ab^	0.01 ± 0.008	0 ± 0	0 ± 0	0 ± 0	0 ± 0^b^	0 ± 0	0.02 ± 0.011
6	1	0.06 ± 0.028^b^	0.03 ± 0.009^c^	0 ± 0^b^	0 ± 0^b^	0 ± 0^b^	0.01 ± 0.007	0 ± 0	0 ± 0	0.01 ± 0.007	0.02 ± 0.009^b^	0.01 ± 0.005	0.02 ± 0.012
Observer													
1	6	0.17 ± 0.054	0.40 ± 0.114	0.09 ± 0.033	0.29 ± 0.127	0.06 ± 0.018^b^	0.01 ± 0.003	0.00 ± 0.00	0 ± 0	0.01 ± 0.004	0.01 ± 0.008	0.01 ± 0.003	0.03 ± 0.010
2	6	0.22 ± 0.055	0.41 ± 0.089	0.08 ± 0.045	0.19 ± 0.097	0.09 ± 0.031^a^	0.01 ± 0.004	0.00 ± 0.00	0.00 ± 0.00	0.01 ± 0.003	0.01 ± 0.004	0.02 ± 0.008	0.02 ± 0.009
Transect type													
Central	5	0.17 ± 0.044	0.41 ± 0.107	0.06 ± 0.032^b^	0.22 ± 0.101	0.06 ± 0.018^b^	0 ± 0	0.01 ± 0.003	0.002 ± 0.001	0 ± 0	0.01 ± 0.006	0.01 ± 0.005	0.03 ± 0.01
Wall	6	0.23 ± 0.067	0.40 ± 0.094	0.12 ± 0.048^a^	0.28 ± 0.129	0.10 ± 0.034^a^	0.01 ± 0.01	0 ± 0	0 ± 0	0.01 ± 0.01	0.02 ± 0.009	0.01 ± 0.008	0.02 ± 0.009
Source of variation		P value											
Flock		< 0.0001	< 0.0001	< 0.0001	< 0.0001	0.0139	0.2618	0.7466	0.5455	0.9423	< 0.0001	0.0920	0.5799
Observer		0.2099	0.4527	0.9219	0.1336	0.0099	0.3469	0.5667	0.3319	0.8990	0.4122	0.1169	0.6475
Transect type		0.1025	0.4520	0.0153	0.9167	0.0246	0.1420	0.0856	0.3238	0.3390	0.1452	0.8125	0.2369

Abbreviation: FL, feather loss.

a-c Values within columns with different letters are significantly different (*P* < 0.05).

### AssureWel Method

The results of the AssureWel assessment showed differences between flocks with regards to minor (score 1) and major (score 2) FL on the back (both *P* < 0.01) as well as somewhat (score 1) dirty birds (*P* < 0.01; Table 7). No birds were observed with dirty areas of plumage larger than 5 cm (score 2) in any flock. Regarding the descriptive flock statistics (Table 8), none of the flocks were beak trimmed. There were between 0 (2 flocks) and 4 (1 flock) antagonistic behaviors observed per flock. Five flocks were evaluated as calm, and one as cautious. There were between 1 and 8 birds per flock identified as needing further care. Based on producer report, total mortality of the previous flock ranged from 2.1 to 4.4%. Mortality of the current flock up to the day of the visit ranged from 0.9 to 2.0%, with missing data for 2 flocks (Table 8).

**Table 7:** Analysis of variance between flocks for the percentage of birds (mean ± SE % of 50 assessed birds/flock) with different degrees of feather loss or dirty plumage according to the AssureWel method^1^.

Flock	FL head (<5 cm)	FL head (>5 cm)	FL back (<5 cm)	FL back (>5 cm)	Dirty (<5 cm)
1	24 ± 6.1	28 ± 6.4	26 ± 6.3^a^	28 ± 6.4^a^	6 ± 3.4^b^
2	6 ± 3.4	0 ± 0	12 ± 4.6^ab^	2 ± 2^b^	30 ± 6.5^a^
3	16 ± 5.2	0 ± 0	26 ± 6.3^a^	32 ± 6.6^a^	0 ± 0^b^
4	0 ± 0	0 ± 0	2 ± 2^b^	0 ± 0^b^	4 ± 2.8^b^
5	18 ± 5.5	0 ± 0	10 ± 4.3^ab^	6 ± 3.4^b^	30 ± 6.5^a^
6	8 ± 3.9	0 ± 0	6 ± 3.4^ab^	0 ± 0^b^	16 ± 5.2^ab^
Source of variation	*P* value				
Flock	0.1539	0.8901	0.0049	0.0060	0.0037

1 FL, feather loss, scored according to the diameter of bare patches.

a-b Values within columns with different letters are significantly different (*P* < 0.05).

**Table 8:** Descriptive presentation of beak trimming, antagonistic behavior, birds needing further care and mortality across flocks according to the AssureWel method.

Flock	Beak trimmed (yes or no)^1^	Antagonistic behavior (n incidents observed)	Flightiness - flock appears calm, cautious, or flighty	Birds needing further care (n)	Mortality previous flock (%)	Mortality to date (%)
1	No	1	calm	1	2.1	unknown
2	No	0	cautious	3	3.1	2.0
3	No	2	calm	3	2.1	0.9
4	No	4	calm	8	Unknown	unknown
5	No	0	calm	2	4.4	2.0
6	No	2	calm	3	3.1	1.6

1 Not permitted in Norway.

### NorWel Method

According to the NorWel method, there were significant differences between flocks with regards to both minor (score 1; *P* < 0.01) and major (score 2; *P* < 0.001) FL on the head. There were also differences in minor and major FL on the back (both *P* < 0.001), minor and major FL on the breast (*P* < 0.001), minor and major FL on the tail (*P* < 0.001) and somewhat (score 1) dirty birds (*P* < 0.01; Table 9). No birds were observed with wounds on the back or tail in any of the flocks. There were no significant differences between transects or tiers in the prevalence of birds with particular welfare indicators (Table 9).

**Table 9:** Analysis of variance between the percentage of birds in each flock (mean ± SE % of 50 assessed birds/flock), in different transects, and different vertical levels with feather loss (severity score 1 and 2) according to the NorWel method^1^.

NorWel	Flocks (n)		FL head (<5 cm)	FL head (>5 cm)	FL back (<5 cm)	FL back (>5 cm)	FL breast (< 5 cm)	FL breast (>5 cm)	FL tail (some)	FL tail (substantial)	Dirty (some)	Dirty (>50% of body)	Wound head
Flock													
1	1		16.5 ± 4.6^ab^	22.3 ± 4.1^a^	33.6 ± 4.8^a^	40.9 ± 4.2^a^	17.5 ± 5.9^a^	21.4 ± 6.3a	36.8 ± 4.9a	42.1 ± 5.2a	0 ± 0b	0 ± 0	0 ± 0
2	1		8.9 ± 3.4^ab^	4.7 ± 3.4^b^	10.9 ± 5.3^bc^	17.2 ± 5.3^b^	3.6 ± 2.5^b^	4.7 ± 3.4^b^	16.1 ± 4.4^ab^	6.3 ± 4.3^b^	20.3 ± 5.3^a^	2.1 ± 2.1	0 ± 0
3	1		0 ± 0^b^	0 ± 0^b^	18.8 ± 6.1^ab^	8.3 ± 4^b^	0 ± 0^b^	0 ± 0^b^	31.8 ± 7.6^a^	7.8 ± 3.8^b^	7.3 ± 5.0^b^	0 ± 0	0 ± 0
4	1		1.3 ± 1.3^b^	4.2 ± 2.8^b^	3.6 ± 2.5^bc^	2.1 ± 2.1^b^	0 ± 0^b^	2.1 ± 2.1^b^	2.1 ± 2.1^b^	0 ± 0^b^	6.5 ± 3.7^ab^	0 ± 0	3.1 ± 3.1
5	1		14.3 ± 5.6^ab^	2.8 ± 2.8^b^	5.6 ± 3.7^bc^	8.2 ± 3.6^b^	2.8 ± 2.8^b^	5.6 ± 3.7^b^	4.9 ± 3.3^b^	4.9 ± 3.3^b^	20.4 ± 5.3^a^	0 ± 0	0 ± 0
6	1		17.4 ± 5.8^a^	1.7 ± 1.7^b^	0 ± 0^c^	2.1 ± 2.1^b^	0 ± 0^b^	0 ± 0^b^	3.8 ± 2.5^b^	0 ± 0^b^	6.9 ± 4.8^ab^	0 ± 0	3.8 ± 2.5
Transect type													
Central	5		9.21 ± 2.569	4.88 ± 1.728	8.57 ± 2.510	10.73 ± 2.563	2.42 ± 1.372	5.02 ± 1.956	10.54 ± 2.487	8.03 ± 2.445	9.88 ± 2.625	0 ± 0	1.02 ± 0.719
Wall	6		7.45 ± 1.841	4.39 ± 1.862	13.65 ± 3.516	11.66 ± 3.160	3.47 ± 1.534	3.47 ± 1.672	20.37 ± 4.309	7.41 ± 2.928	12.17 ± 3.321	0.92 ± 0.925	1.38 ± 1.031
Vertical level													
1 (litter)	6		9.5 ± 3.3	7.9 ± 3.4	8.5 ± 3.4	15.6 ± 4.6	2.1 ± 1.5	8.5 ± 3.6	12.3 ± 4.1	10.8 ± 4.6	9.8 ± 3.6	0 ± 0	0 ± 0
2 (1st tier)		6	10.8 ± 4.2	3.9 ± 1.8	8.8 ± 4	13.1 ± 4.4	3.3 ± 2.3	2.1 ± 1.5	14.8 ± 4.5	8.8 ± 4.2	16.5 ± 4.9	1.7 ± 1.7	2.3 ± 1.6
3 (2nd tier)	6		3.8 ± 2.1	3.3 ± 1.9	13.3 ± 4.9	5.2 ± 2.5	1.7 ± 1.7	4.2 ± 2.4	17.1 ± 6.2	6.7 ± 3.3	10.8 ± 4.6	0 ± 0	0 ± 0
4 (top tier)	6		9.7 ± 3.1	3.5 ± 2.6	12.8 ± 4.6	10.8 ± 4	4.5 ± 2.6	2.5 ± 2.5	15.8 ± 4.6	4.8 ± 2.8	6.7 ± 3.2	0 ± 0	2.5 ± 2.5
Source of variation								*P* value					
Flock			0.0018	< 0.0001	0.0003	< 0.0001	< 0.0001	0.0002	< 0.0001	< 0.0001	0.0262	0.5874	0.4389
Transect type			0.9321	0.6535	0.3576	0.9263	0.6727	0.4144	0.0546	0.5327	0.4689	0.3159	0.7328
Vertical level			0.3429	0.4035	0.7031	0.1152	0.6643	0.1796	0.8520	0.4320	0.3599	0.4043	0.4666

1 FL, feather loss, scored as diameter of bare patches or, for tail, severity of wear.

a-c Values within columns with different letters are significantly different (*P* < 0.05).

### Correlations Between Welfare Assessment Results From the Three Different Methods

The Aviary Transect, AssureWel and NorWel methods included different welfare indicators and scores that were not comparable. However, 3 welfare indicators from each method were considered comparable across methods: FL on the head and back, and dirtiness (Table 10). Scoring of all 3 indicators by the Aviary Transect method showed good agreement with both AssureWel and NorWel (Table 10). Scoring of FL on the head also showed good agreement between AssureWel and NorWel (Table 10).

**Table 10:** Correlations between feather loss on head and back, and dirtiness, as assessed by three different methods – Aviary Transect, AssureWel, and NorWel.

	FL Head^1^			FL Back^2^			Dirty^3^		
	Aviary Transect	AssureWel	NorWel	Aviary Transect	AssureWel	NorWel	Aviary Transect	AssureWel	NorWel
Aviary Transect	1	0.891*	0.954⁎⁎	1	0.857*	0.892*	1	0.855*	0.838*
AssureWel		1	0.978⁎⁎⁎		1	0.558		1	0.580
NorWel			1			1			1

⁎ *P* < 0.05.

⁎⁎ *P* < 0.01.

⁎⁎⁎ *P* < 0.001.

1 Defined as missing feathers on >5 cm diameter patch of head by all three methods.

2 Defined as missing feathers on >50% of back including wings by Aviary Transect method, and >5 cm diameter by AssureWel and NorWel.

3 Defined as dirt covering >25% of plumage by Aviary Transect method, dirt covering <5 cm by AssureWel, and some dirt by NorWel.

## DISCUSSION

The aim of this study was to investigate differences in welfare assessment results for flocks of laying hens in aviaries according to 3 different approaches: an Aviary Transect method, AssureWel, and a method used by Norwegian farm advisors called NorWel. As the Aviary Transect method is new, we also measured the time required, interobserver reliability, and within- and across-house sensitivity of this method. All flocks in the study were commercial flocks of white-strain hens, with similar flock size and bird age, kept in multitiered aviary systems under standardized management (KSL, 2020). Despite this relative homogeneity, all 3 methods detected differences between flocks for several of the assessed welfare indicators.

The Aviary Transect method was based on the routine checks performed daily by egg producers combined with the established methodology from broiler and turkey transect walks (Marchewka et al., 2013, 2015; BenSassi et al., 2019a,b,c). When applying transects sampling methodology, the entire flock is observed, and the frequency of birds falling within each predefined welfare indicator are scored (Marchewka et al., 2013). This places the focus on the more severe cases, improves observer agreement and reduces the risk of omitting birds (D'Eath et al., 2012; Main et al., 2012; Marchewka et al., 2015). The 12 animal-based welfare indicators included in the Aviary Transect assessment were selected based on their relevance for laying hen welfare (Tauson et al., 2005; Blokhuis et al., 2007; Rodenburg et al., 2008, Welfare Quality®, 2009) and strong interobserver reliability (Decina et al., 2019). The Aviary Transect method detected significant variation across flocks for 6 of the 12 welfare indicators: FL on the head, back, breast, and tail, dirty birds, and enlarged crop. For most of the other indicators, the incidence was very low for the observed flocks, thus lacking enough variability to detect differences between them. In comparison, AssureWel included 8 animal-based welfare indicators and significant differences between flocks were detected for 2 of them, FL on the back and dirtiness. Of NorWel's 8 animal-based welfare indicators, we found significant differences between flocks for 5 of them, FL on the head, back, breast, and tail, and dirty birds.

All three methods allowed the detection of flock differences in plumage condition. Plumage condition generally deteriorates with age (Rørvang et al., 2019), but the main reason for poor plumage was likely feather pecking (Rodenburg et al., 2013). Feather pecking is a detrimental behavior in poultry that causes pain for the victim (Bright, 2008), increased mortality (Heerkens et al., 2015), and economic losses for the producers as hens with poor plumage increase their feed intake to compensate for heat loss (Glatz, 2000). In welfare assessment schemes, feather scores are typically collected for different body parts and then summed to give an overall score (e.g., Welfare Quality®, 2009). However, some risk factors only involve damage to specific body regions (Campe et al., 2018). For example, damaging feather pecking is directed mainly at the back and vent area (Bilcik and Keeling, 1999; Heerkens et al., 2015) while feather damage to the head and neck can be due to abrasion (Blokhuis et al., 2007). The specific scores for different body parts collected using the three methods evaluated in the current study provide producers with quantitative data that facilitate the pinpointing of specific welfare problems that need to be addressed.

With regards to scoring of FL on the head (>5 cm), the transect scores ranged from 0.03 to 0.81% of the birds, while the corresponding ranges for AssureWel and NorWel were 0 to 28% and 0 to 22.3%, respectively. Despite these inherent differences, the results from the Aviary Transect method were highly correlated with the results from AssureWel and NorWel in the observed flocks. In fact, in 2 of 3 comparable indicators, agreement between the Aviary Transect method and each of the other 2 methods was better than that between AssureWel and NorWel. As the latter 2 methods scored FL on the head and back using identical categories (score 1: <5 cm, score 2: >5 cm) on 50 random birds, we could expect a high level of agreement between them. This was the case for FL on the head but not the back, suggesting that the whole-flock scoring done in the Aviary Transect method gives a more reliable assessment of FL in large flocks.

When there is a large variation between birds in a flock, a larger sample size provides a more reliable estimate of prevalence (Bright et al., 2006). This may especially be true for less common welfare issues such as wounds, which were rarely observed in the studied flocks. The Aviary Transect method detected wounds on the head and back in 3 of the flocks, and wounds on the tail in one flock. Wounds are not specified as a welfare indicator in AssureWel. Using the NorWel method, we detected wounds on the head in 2 flocks, but no wounds on back or tail. Overall, these results suggest that the Aviary Transect method may be more sensitive compared to sampling a fixed number of individuals for important welfare issues with low flock prevalence. Visual assessment of the entire flock is considered time consuming but, in our study, the 3 methods all took approximately 20 min to complete.

Individual sampling is a common method for assessing laying hen welfare, including observing 50 birds from a distance (AssureWel, LayWel) or catching 150 birds for inspection (Welfare Quality®, 2009). The Aviary Transect, AssureWel, and NorWel methods comprise partly different welfare indicators and categories, making it difficult to compare the methods directly. Another issue is the difference in sample size. For instance, one bird scored as dirty out of 50 assessed birds will result in a 2% prevalence whereas one bird scored as dirty in transect sampling will indicate a lower prevalence because all the birds within each transect are assessed (Marchewka et al., 2013). The transect method delivered a similar range of values as for the welfare indicators found in broilers and turkeys (Marchewka et al., 2015). Thus, the range of values obtained with the Aviary Transect method may be more realistic as compared with the high prevalence rates found in some flocks with the AssureWel or NorWel methods. Scoring a limited sample of individuals in herds of large animals such as cows may provide a reliable estimate (Ito et al., 2009) but the same approach in flocks of thousands of animals may be less reliable.

The transect method has been shown to be an effective tool for early detection to predict welfare issues and production results in broilers (BenSassi et al., 2019a,b) and turkeys (Marchewka et al., 2019, 2020; Vasdal et al., 2021). As production cycles in laying hens last longer, application of the Aviary Transect method at an early production stage may be even more valuable, as emerging issues will cause larger problems over longer production periods (Drake et al., 2010). The associations between early assessments using the Aviary Transect method and later welfare and production results in laying hen flocks should be the focus of further studies.

The Aviary Transect method scores birds in each transect, and the scores can then be combined to give an overall flock score. For most indicators, there were no differences in prevalence between transects, suggesting that birds with these issues are evenly distributed around the house. However, we found more laying hens with FL on the breast and dirtier birds in wall transects as compared to central transects. This result differs from findings in broiler flocks, where a higher prevalence of sick birds (BenSassi et al., 2019a) or dead birds (Marchewka et al., 2013) was observed in wall transects compared to central transects. Sick birds are less mobile, and once they are close to the wall, they are less likely to move away (Estevez and Christman, 2006; BenSassi et al., 2019c), but similar effects have not been reported for aviary-housed laying hens, possibly due to their typically lower mortality rate and presence of tiers and nests where sick birds may rest. In a recent study, hens with keel bone fractures, a common welfare issue in laying hens, were reported to spend more time on the upper tiers and less time in the litter area (Rufener et al., 2019).

The transect method showed very good interobserver agreement when applied in aviaries, with dirty birds being the only indicator that differed significantly between observers. This is in accordance with results reported by Marchewka et al. (2013), where scoring of dirty broilers differed significantly between observers. Assessing dirtiness might be more influenced by the lighting conditions compared to other indicators (Marchewka et al., 2013). Dirtiness was also one of the least clear-cut indicators in the Aviary Transect method (<25% of the plumage). Further studies should investigate if improved observer training and a more detailed description of this indicator can reduce interobserver variation.

On-farm welfare assessment is challenging because it needs to be noninvasive, adaptable to different farms, and cost-efficient (Marchewka et al., 2013). It must also be based on validated welfare indicators and reliable methods. Future studies should provide further evidence to ensure that the Aviary Transect method provides a reliable assessment of the welfare status across a range of aviary lay-outs, flock sizes, ages, and strains of laying hens. Another area for research is the potential simplification of the method, as previous studies in broilers suggest that it is, in fact, sufficient to assess only part of the house, comprising one wall transect and one central transect (BenSassi et al., 2019c).

In conclusion, the present results show that all 3 methods were time efficient and detected differences between flocks for several of the assessed welfare indicators. The AssureWel and the NorWel methods gave graded scores for 8 welfare indicators, providing information about the severity of each indicator, but on relatively few animals. In contrast, the newly developed Aviary Transect method evaluated every bird in the flock for 12 indicators on a binary scale, resulting in a relatively sensitive evaluation of the prevalence of each indicator, with a focus on the more severe cases in the flock. Thus, the Aviary Transect appears to be an efficient and sensitive whole-flock assessment method for laying hens in multitiered aviary systems. The transect method has good interobserver reliability and good agreement with methods involving graded sampling of a subset of individuals, with the added benefit of including a higher number of welfare indicators. The Aviary Transect method has the potential to provide egg producers with a quantitative and specific assessment of the welfare status in the flock that is easy to use on the farm.
